# Wild Rabbit Exposure to *Leishmania infantum*, *Toxoplasma gondii*, *Anaplasma phagocytophilum* and *Babesia caballi* Evidenced by Serum and Aqueous Humor Antibody Detection

**DOI:** 10.3390/microorganisms9122616

**Published:** 2021-12-17

**Authors:** Labrini V. Athanasiou, Eleni G. Katsogiannou, Constantina N. Tsokana, Sofia G. Boutsini, Marina G. Bisia, Vasileios G. Papatsiros

**Affiliations:** 1 Department of Medicine, Faculty of Veterinary Medicine, University of Thessaly, 43100 Karditsa, Greece; elkatsog@uth.gr (E.G.K.); kotsokan@vet.uth.gr (C.N.T.); mbisia@outlook.com (M.G.B.); vpapatsiros@vet.uth.gr (V.G.P.); 2 Veterinary Centre of Athens, General Directorate of Veterinary Services, Parasitology—Parasitic Diseases, Entomology and Bee Health Department, 15341 Athens, Greece; sboutsini@yahoo.gr

**Keywords:** aqueous humor, *A. phagocytophilum*, *B. caballi*, *L. infantum*, *T. gondii*, antibodies, Indirect Immunofluorescence Antibody Assay, serology, wild rabbits

## Abstract

Wild rabbits (*Oryctolagus cuniculus*) can be important sentinel species for the presence of zoonotic pathogens. Therefore, we collected blood samples from wild rabbits harvested by hunters during the hunting season 2019–2020 on the island of Lemnos, to determine exposure of wild rabbits to the zoonotic pathogens *Leishmania infantum*, *Toxoplasma gondii*, *Anaplasma phagocytophilum* and *Babesia caballi*, as well as aqueous humor to assess its diagnostic performance in terms of sensitivity, specificity, positive and negative likelihood ratios. Antibodies against these pathogens were detected by Indirect Immunofluorescence Antibody (IFA) assay. Out of the 72 wild rabbits included in the study, 4.2%, 5.5%, 18% and 9.7% were seropositive to *L. infantum*, *T. gondii*, *A. phagocytophilum* and *B. caballi*, respectively. Although less frequently, antibodies were also detected in aqueous humor of wild rabbits. The antibody detection in aqueous humor presented 100% specificity but decreased sensitivity compared to serum suggesting that aqueous humor could be successfully used in epidemiological studies to confirm exposure at the population level but has little diagnostic value at the individual level. This is the first report on the seropositivity of wild rabbits to *A. phagocytophilum* and *B. caballi* and the detection of antibodies against *A. phagocytopylum*, *L. infantum*, *T. gondii* and *B. caballi* in the aqueous humor.

## 1. Introduction

*Oryctolagus cuniculus* is one of the most widespread lagomorph species occupying a huge variety of ecosystems. Their intermediate size and great abundance allows them to support a community of small to medium-sized predators such as foxes, cats and civets with which they share the same habitats. This species occurs in both wild and domestic forms. Its domestic counterpart is raised globally for meat, wool and fur, and it is also an increasingly popular pet [1]. Besides, they are considered to be a useful sentinel species for the level of environmental contamination and the circulation of pathogens in their habitat [2,3].

Leishmaniosis caused by the protozoan *Leishmania infantum* is a severe vector-borne zoonotic disease that is endemic in the Mediterranean basin [4]. Transmission occurs via the bite of female sand flies of the subfamily *Phlebotominae*. *L. infantum* is the causative agent of zoonotic visceral (VL) and cutaneous leishmaniosis (CL) in humans, and of canine leishmaniosis in dogs, the main reservoir host of the parasite [5]. The existence of a sylvatic *L. infantum* transmission cycle in wildlife that overlaps with the domestic cycle maintained by dogs has been well documented as one of the main factors limiting disease control [6,7]. The outbreak of human leishmaniosis in southern Madrid involving Iberian hares (*Lepus granatensis*) and wild rabbits (*O. cuniculus*) [7,8] as well as the following studies in *Lepus europaeus* in different European countries including Greece [9,10,11] showed how anthropogenic interventions in ecosystems that lead to high density of some wild species, and increased contact with domestic animals and humans, could dramatically affect the epidemiology of a disease [12,13]. Importantly, rabbit infectiousness to *P. perniciosus* sand flies has been demonstrated by xenodiagnosis suggesting the competence of this species as a host reservoir [8].

*Toxoplasma gondii*, a protozoan parasite with global distribution, can infect virtually every warm-blooded animal, including humans and livestock which act as intermediate hosts. Domestic and wild felids are the definitive hosts, being able to excrete oocysts to the environment. Humans commonly get infected through consumption of undercooked or raw meat containing tissue cysts [14]. However, ingestion of oocysts directly from the environment, indirectly via contaminated food or drinking water or while field dressing game by hunters, can also lead to infection [14,15]. As for the lagomorphs, they are mainly infected with *T. gondii* via the ingestion of water and plants contaminated with oocysts excreted by felids with which they share the same habitats [16]. Thereafter, infected lagomorphs can act as a potential source of *T. gondii* for other animals, especially for their predators, but also for humans [16,17].

Concerning the *Anaplasma* species, obligate rickettsial pathogens, that are mainly transmitted by different species of hard ticks and proliferate inside red blood cells, cause clinical and subclinical infections in a variety of vertebrate hosts. To the authors’ knowledge, there are no data available in the literature on the *Anaplasma* spp infection/exposure status of *O. cuniculi*. However, there is increasing evidence of *A. phagocytophilum* infection/exposure in several wild rabbit species including *Le. europaeus* in Italy [18], cottontail rabbits (*Sylvilagus floridanus*) in Massachusetts [19,20], *Lepus sinensis* in China [21] and riparian brush rabbits (*Sylvilagus bachmani riparius*) in California [22].

Babesiosis is a vector-borne disease caused by erythrocytic protozoal parasites of the genus *Babesia*. These piroplasms are of medical and veterinary importance worldwide and can cause severe disease in humans, domestic animals and wildlife [23,24]. Transmission of *Babesia* spp. mainly occurs through the bite of infected ixodid ticks [25]. In Europe, *B*. *divergens* remains the most frequent cause of human babesiosis, while in North America, *B*. *microti* is the most reported species associated with human disease. Other zoonotic species that have been reported are *B*. *duncani* and *B*. *venatorum* [26,27]. *B. caballi* is the aetiological agent of equine piroplasmosis, a tick-borne disease, which is characterized by persistent infection and carriers act as sources of infection for ticks [28,29].

Blood sampling can be challenging in the context of seroepidemiological studies in wild animals [30]. Regarding carcasses, blood collection through right heart puncture may not be successful due to post mortem blood clotting. In recently deceased animals whose blood has not yet clot, further handling is needed after blood is drawn. Blood samples should be centrifuged or left undisturbed for approximately 30 min to encourage clot formation [31,32]. Besides, it has been suggested that after death, blood rapidly deteriorates due to post mortem blood clotting, contamination by bacteria, release of intracellular chemicals and metabolism of serum compounds [32]. Thus, the collection of samples alternative to blood and serum, like blood on filter papers or aqueous humor, have been evaluated in terms of their diagnostic accuracy in different animal species and for several pathogens [30,33,34,35,36,37,38,39].

Aqueous humor is an eye-specific sample, easy to obtain, with good stability and minimally invasive compared to other types of eye specimens [40,41,42]. Recently, it has gained attention as an alternative biological sample that could assist in the diagnosis of several infectious and neoplastic ocular diseases in human and animal medicine. Thus, a number of studies used serologic, molecular and immunocytochemical assays as well as cytologic examination to define the diagnostic efficiency of aqueous humor in dogs, cats and rabbits [37,43,44,45].

The diagnostic utility of aqueous humor for the detection of antibodies in rabbits has been previously investigated only for anti- *Toxocara canis* and anti- *T. gondii* IgG following experimental infection [33,38]. To the authors’ knowledge, there are no data available coming from field studies in naturally infected rabbits. Moreover, data on the exposure rate of wild rabbits in Greece to pathogens with zoonotic potential, is limited to one study concerning *L. infantum* infection [46]. This is especially important for the wild rabbit population in the island of Lemnos, northern part of the Aegean Sea, Greece, where since 1995, wild rabbits, due to overpopulation, have significantly disrupted the ecosystem, causing huge losses and extensive damage to crops. Significant effort has been made by the state authorities for the management of rabbit population in the island [47,48,49]. Thus, the objectives of this study were (a) to provide evidence on the occurrence of wild rabbit exposure to *L. infantum*, *T. gondii*, *A. phagocytopylum* and *B. caballi* in the island of Lemnos, northern part of the Aegean Sea, Greece (b) to assess the diagnostic utility of aqueous humor for the detection and quantification of IgG antibody levels against the above-mentioned pathogens compared to serum samples in naturally infected wild rabbits.

## 2. Materials and Methods

### 2.1. Animals

The samples included in this study were collected from 72 wild rabbits from the island of Lemnos (Longitude: 25°11′45.38″ E, Latitude: 39°56′35.77″ N) which were hunter harvested during the hunting season 2019–2020 according to the prerequisites of the Greek Legislation (Hellenic Government Gazette 3137/6-8-2019, issue B) [50]. The island of Lemnos is located in the northern part of the Aegean Sea covers an area of 477.583 square kilometers, has Mediterranean climate and strong winds. The authors declare that no animals were killed for the purpose of this study and that all procedures contributing to this work met the ethical standards of the relevant national and European regulations on the care and use of animals (Directive 2010/63/EC).

### 2.2. Sampling

Paired blood and aqueous humor samples were collected within three hours from the death of animals. More specifically, blood samples were collected from the heart, transferred into sterilized containers and an average of 0.3 mL of aqueous humor was collected with gently aspiration from both eyes using a syringe with a 21G needle, which was inserted horizontally just under the cornea into the anterior chamber. The aqueous humor and blood samples were transferred to the laboratory. Blood samples were centrifuged at 400× *g* for 10 min for serum recovery. All samples were stored at −20 °C pending analysis.

### 2.3. Indirect Immunofluorescence Antibody (IFA) Assay

All the serum and aqueous humor samples were tested, by indirect fluorescence antibody test (IFAT), for the presence of the antibodies against *L. infantum*, *T. gondii*, *A. phagocytophilum* and *B. caballi* using different commercial agent specific slides (Fluoleish, Biovetotest Diagnostic Veterinaire, France, Fuller Laboratories Fullerton, California, USA, MegaFLUO^®^ ANAPLASMA ph. Horbranz, Austria and Agrolabo, Scarmagno, Italy, for *L. infantum*, *T. gondii*, *A. phagocytophilum* and *B. caballi*, respectively). For all IFATs, a fluorescein isothiocyanate conjugated anti-rabbit IgG (Sigma-Aldrich, St Luis, MO, USA) was used.

For the detection of antibodies, in both serum and aqueous humor, against *L. infantum*, *T. gondii* and *A. phagocytophilum*, a dilution of 1:25 was used as cut off value, while the threshold value for the detection of antibodies against *Babesia* spp. was 1:50, as previously reported [22,51,52,53]. Moreover, a cut off value of 1:10 was used in aqueous humor, for the detection of antibodies against all the above-mentioned microorganisms. A Nikon Eclipse E-400 fluorescence microscope was used for the observation (objective × 100).

### 2.4. Data Analysis

For the statistical analysis of the data, MedCalc Statistical Software v.14.8.1 (MedCalc Software bvba, Ostend, Belgium; http://www.medcalc.org; 2014, accessed on 10 November 2021) was used in order to calculate the sensitivity, the specificity, Positive likelihood ratio (PLR) and Negative likelihood ratio (NLR). PLR values >10 and NLR values <0.1 are indicative of good test performance [54]. Moreover, the agreement between the results of the tests performed in the two different biological samples was measured using the Cohen’s Kappa (κ) value. A value of 0 indicates fair agreement, while a value of 1 indicates a perfect agreement [55,56]. A value of *p* ≤ 0.05 was considered significant in all comparisons.

## 3. Results

**Figure 1:**
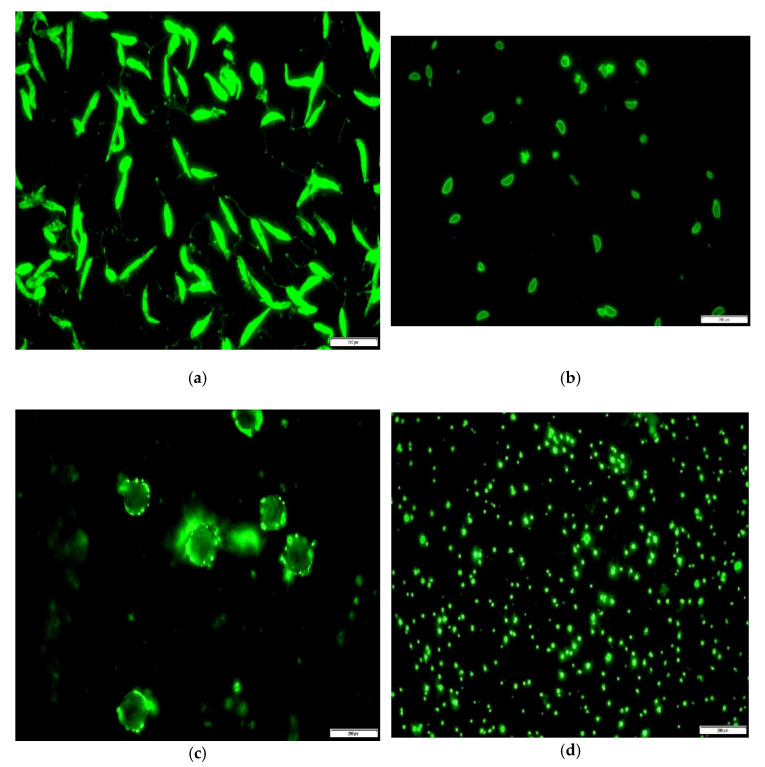
Image of Indirect Immunofluorescence Antibody (IFA) assay observed by a Nikon Eclipse fluorescence microscope (objective × 100). (**a**) *L. infantum* positive IgG antibody reaction, serum titer 1:25; (**b**) *T. gondii* positive IgG antibody reaction, serum titer 1:25, (**c**) *A. phagocytophilum* positive IgG antibody reaction, serum titer 1:25 and (**d**) *B. caballi* positive IgG antibody reaction, serum titer 1:50.

Out of the 72 serum samples tested, three (4.2%) were positive against *L. infantum*, four (5.5%) against *T. gondii*, 13 (18%) against *A. phagocytophilum* and seven (9.7%) against *B. caballi* (Figure 1). Employing the same cut off values in aqueous humor examination, resulted in fewer positive samples; one (1.3%) for the detection of antibodies against *L. infantum* and *T. gondii*, four (5.5%) and two (2.7%) against *A. phagocytophilum* and *B. caballi*, respectively. When the cut off value of 1:10 was selected, equal number of positive samples was obtained for *L. infantum* and *T. gondii* and higher for the other two microorganisms (6 and 3, respectively) (Table 1).

The results of antibody detection against *L. infantum*, *T. gondii*, *A. phagocytophilum* and *B. caballi* in wild rabbits, in serum and in aqueous humor at individual level using different cut-off points are presented in the Supplementary Table S1.

The agreement between the serum and the aqueous humor samples examination, as it is shown by the Cohen’s Kappa (κ) values, was low when the same cut off value was used (1:25 or 1:50) for both types of samples. However, in the case of *A. phagocytophilum* and *B. caballi*, a better agreement was achieved when a cut off value of 1:10 was used for the aqueous humor (Table 2). This improvement in κ values was probably evident only for these two pathogens due to the higher number of positive samples detected.

As for the diagnostic accuracy of antibody detection in aqueous humor compared to serum, which was used as the reference standard, the sensitivity, specificity, positive and negative likelihood ratios were calculated and they are presented in Table 3 and Table 4, for the same with serum and the 1:10 cut off values, respectively. More specifically, the highest sensitivity was observed for anti-*L.infantum* antibodies when the cut-off value applied was 1:10 (60%). The specificity was almost perfect in all cases regardless of the cut-off value used. PLR values showed good performance in all cases while the best NLR value (lower) was observed for anti-*L.infantum* antibodies when the cut-off value applied was 1:10.

## 4. Discussion

Wild rabbits are important small game animals extensively hunted in many countries. Rabbit meat is considered one of the most nutritional white meats and the demand for human consumption of rabbit meat is increasing. Apart from its economic significance, domestic rabbits are also scientifically important as key laboratory animals in medical research. On the other hand, rabbits are pests of national significance in several geographical areas [1,57]. These aspects together with the abundance of wild rabbits and their close proximity to humans and domestic animals have resulted in increasing scientific interest and research, including the occurrence of pathogens with zoonotic potential in rabbit populations globally.

In the present study, wild rabbit samples were screened for the presence of antibodies against *L. infantum*, a zoonotic disease steadily endemic in canine population in Greece [58,59]. Serological data in wild rabbits in Greece come from a previous study conducted in a wild rabbit population in the island of Lemnos and domestic rabbits from three Regional Units of Central Macedonia (Thessaloniki, Chalkidiki, Serres). This study showed an overall seroprevalence of 7.6% in domestic and wild rabbits [46]. A low percentage of seropositivity was observed in the wild rabbit population however, in agreement to the results of the present study. Similarly, serological studies in wild rabbits showed a variance of 0%–75.4% leading to controversial conclusions on the possible role of rabbits in the epidemiology of leishmaniosis [46,51,60,61,62]. A recent study confirmed that natural *Leishmania* infection in wild rabbits is not associated to gross pathology and only minimal histopathological lesions were observed while *L. infantum* antigens were most frequently detected in skin [51,63]. Previous studies showed that *L. infantum* is widely spread in wild rabbit populations with infection prevalence ranging from 0% to 100% [2,46,51,60,61,64,65,66]. Highly heterogeneous prevalence values were recently reported even in different municipalities in Spain, suggesting that apart from the intrinsic restrictions of the methods applied and the samples selected for examination, *Leishmania* infection is clustering in space and time in local scale [2]. When optimal circumstances exist in terms of co-existence and close proximity of competent host reservoirs and vectors in a geographical area, the sylvatic and domestic transmission cycle of *L. infantum* may overlap [6].

Different species of warm-blooded mammals, birds, as well as human [14,67] comprise the wide range of hosts that have been found exposed to *T.gondii*. Several studies have shown the occurrence of antibodies against *T.gondii* in rabbits, mainly the European rabbit (*O. cuniculus*) with seroprevalences ranging from 0.9% to 37.5% [17,68,69]. In wild rabbits, low seroprevalences of 3.3% in Scotland [70] and 2.8% in Portugal [71] were reported while a study in Australia showed that the mean seroprevalence was 9.9% [3] and in Spain a seroprevalence as high as 53.8% was previously recorded [52]. Compared to the above-mentioned studies, the seropositivity of 5.5% detected in the present study, is considered as quite low. High prevalence of infection has been reported in Portugal (67.9%) [72] and Mexico (68.4%) [73]. Infections in rabbits are mainly subclinical [16,70]. However, viable *T. gondii* could still be present in the tissues of seropositive lagomorphs posing a threat for hunters while handling apparently healthy individuals in the field [70].

This is the first report, to the authors’ best knowledge, regarding the presence of antibodies against *A. phagocytophilum* and *B. caballi* in wild rabbits, although, antibodies against these tick-borne pathogens have been identified in other hosts. As for *A. phagocytophilum*, the pathogen has been found in humans [74] and in certain animal species [75,76], in Greece. However, recently in a laboratory study aiming to assess the vector competence of *Rhipicephalus sanguineus* for *Anaplasma platys*, previously naïve New Zealand white rabbits, which are not known to be susceptible to *A. platys* infection, produced IgG antibodies detectable in IFAT [77]. Moreover, increasing evidence of *Anaplasma* infection in lagomorphs exists, with *A*. *phagocytophilum* being the most frequently reported in other wild rabbit species with seroprevalence ranging from 0.9% in *Le. europaeus* in Italy [18] to 66% in cottontail rabbits (*Sylvilagus floridanus*) in Massachusetts [19,20] and DNA prevalence ranging from 1.86% in *Le. sinensis* in China [21] to 29% in riparian brush rabbits (*Sylvilagus bachmani riparius*) in California [22]. No evidence of obvious infection has been recorded so far while it has been suggested that wild rabbits may act as maintenance hosts. However, this hypothesis needs further elucidation. Public health concern is raised in this case due to the occurrence of infected wild rabbits in close proximity to humans and on the propensity of the involved ticks to attach and feed on humans and other vertebrate hosts [78].

To the authors’ best knowledge, there is no evidence of *Babesia* spp. infection in *O.cuniculus* in the literature. In this study, anti-*B. caballi* antibodies have been detected in 9.7% of *O.cuniculus* using IFAT in serum samples. *B. caballi*, the causative agent of equine piroplasmosis has never been reported in rabbits. However, as it has been shown in previous studies, cross reaction between different *Babesia* spp., including *B. caballi*, cannot be excluded [28,29]. Zoonotic *Babesia* spp have been detected in other wild rabbit species. Interestingly, *B. divergens* DNA was detected in 16% of the Eastern cottontail rabbits (*S. floridanus*) sampled in Massachusetts [79]. *B. divergens* is vectored by *Ixodes dentatus*, a rabbit and bird feeding tick that may also feed on human. Molecular evidence indicated that the same *Babesia* spp. was identified in human cases in Missouri and Kentucky [79], suggesting that this species may be a reservoir hosts for the parasite. A single eastern cottontail rabbit was molecularly positive for *Babesia* sp. MO1 in Tennessee, while in the same study, 25% of the rabbits were found seropositive for *B. odocoilei*, 38% for *Babesia* sp. MO1 and 25% for both *B. odocoilei* and *Babesia* sp. MO1, suggesting possible cross-reaction or, potentially, co-infection [80]. In Greece, *Babesia* spp. has been serologically, cytologically and/or molecularly identified in horses [81], ruminants [82,83] and dogs [75].

The presence of antibodies in the aqueous humor can be attributed to either an increased permeability of the blood aqueous barrier or to the local antibody production, especially caused by microorganisms involved in the pathogenesis of uveitis [84,85]. Moreover, the correlation between the protein concentration of aqueous fluid and plasma depends on the molecular weight of the protein and it is reported to be stronger for small proteins [86].

In the present study, we report for the first time the detection of antibodies against *L. infantum*, *T. gondii*, *A. phagocytophilum* and *B. caballi* in the aqueous humor of naturally infected wild rabbits. The presence of *T. gondii* antibodies in aqueous humor of rabbits was shown previously in an experimental study conducted to investigate the local production of IgG in ocular toxoplasmosis using a rabbit model of the disease. This study suggested that specific antibodies were detectable and persistent in aqueous humor and serum for at least 100 days [38]. Another study in rabbits experimentally infected with *Toxocara canis*, showed that anti-*T. canis* IgG levels in the aqueous humor were well correlated with the severity of intraocular inflammation. The authors suggested that the discordance between serum IgG and aqueous humor IgG levels cannot be explained solely by the diffusion of parasite-specific antibodies through a leaky blood–aqueous barrier but also by the local antibody production in the eye [33]. Concerning the anti-*Leishmania* IgG detection in aqueous humor, previous studies showed the presence of higher IgG levels in ocular sampled of dogs with uveitis despite the level of antibody in the serum or even in the absence of anti-*Leishmania* IgG in the serum [37,87]. In the latter study, positive results were obtained from the aqueous humor and plasma samples in 72% of the dogs and a C value greater than one was observed in 56% of the studied animals. The authors suggested that the anti-*Leishmania* antibody levels in plasma were superior to those found in the aqueous humor [87]. In the present study, lower IgG levels were found in the aqueous humor of naturally infected rabbits compared to serum, for all the pathogens examined, especially when the same cut off value was used in both biological samples.

Despite the uncertainty of the origin of the antibodies in the present study, as well as in previous studies, the assessment of the diagnostic utility of the ocular fluid revealed that the detection of antibodies in aqueous humor presents high specificity (100%) but low sensitivity compared to the antibody detection in serum suggesting that aqueous humor could be successfully used in epidemiological studies to confirm exposure at population level but has little diagnostic value at the individual level. In the present study, the diagnostic utility of the ocular fluid was assessed by sensitivity and specificity while the positive and negative predictive value were not reported, since they depend on the prevalence of the disease which for the pathogens tested in this study, was unknown [54]. To the contrary, both PLR and NLR were calculated which are independent of disease prevalence and invariable among different populations or settings. The PLR, however, could be calculated only when antibodies against *A. phagocytophilum* and *B. caballi* were detected in the ocular fluid with a cut off value of 1:10, whereas in all other cases, this was not possible because the 100% specificity resulted in the denominator of the equation to be zero. Based on the PLR value, which in all cases was indicative of good performance, antibodies are likely to be detected in the ocular fluid for a seropositive animal. To the contrary, the NLR values are indicative of poor performance, while the lowest (best) NLR value was calculated for the detection of antibodies against *L. infantum* using a cut off value 1:10.

Moreover, the agreement between the results of antibodies in both fluids were fair to moderate and it was improved when the 1:10 cut off value was used in the aqueous humor. However, further studies in a larger number of samples are needed for the establishment of the optimal cut-off value to assure the best combination of specificity and sensitivity values in aqueous humor. Additional studies are also required to determine the real prevalence of these pathogens and the role of wild rabbits in their epidemiology such as molecular studies for the detection of the pathogens in organ/meat samples.

## 5. Conclusions

Exposure of wild rabbits to *L. infantum*, *T. gondii*, *A. phagocytophilum* and *B. caballi* in Greece was evidenced by the detection of antibodies against all the above-mentioned pathogens in serum as well as in aqueous humor. The lower IgG levels in ocular fluid for all the pathogens included in the study compared to serum, is suggestive of employing a low cut off value to increase sensitivity of the antibody detection in the aqueous humor. This study showed that negative results in the ocular fluid do not preclude the possibility of the presence of antibodies in the serum samples of wild rabbits. On the other hand, antibody detection against *L. infantum*, *T. gondii*, *A. phagocytophilum* and *B. caballi* in the ocular fluid almost certainly indicate the presence of antibodies against each pathogen in serum suggesting that aqueous humor could be successfully used in epidemiological studies to confirm exposure at population level but has little diagnostic value at the individual level.

## Figures and Tables

**Table 1:** Number and percentage of positive samples for antibodies against *L. infantum*, *T. gondii*, *A. phagocytophilum* and *B. caballi* in wild rabbits, in serum and in aqueous humor, with the same cut off value (1:25 or 1:50) and with a lower cut off value (1:10) in aqueous humor.

	Serum			Aqueous Humor		
	Cut Off Value	Positive		Cut Off Value	Positive	
		N	%		N	%
*L. infantum*	1:25	3	4.2	1:25	1	1.3
				1:10	1	1.3
*T. gondii*	1:25	4	5.5	1:25	1	1.3
				1:10	1	1.3
*A. phagocytophilum*	1:25	13	18	1:25	4	5.5
				1:10	6	8.3
*B. caballi*	1:50	7	9.7	1:50	2	2.7
				1:10	3	4.2

N: number of positive samples, %: % positive samples.

**Table 2:** Agreement between serum and aqueous humor of wild rabbits for the detection of antibodies against *L. infantum*, *T. gondii*, *A. phagocytophilum* and *B. caballi*.

	S-AH1	S-AH2
*L. infantum*	0.489	0.489
*T. gondii*	0.386	0.386
*A. phagocytophilum*	0.421	0.584
*B. caballi*	0.419	0.575

S: serum, AH1: aqueous humor with the same cut off value with the serum, AH2: aqueous humor with a cut off value of 1:10.

**Table 3:** Sensitivity, specificity and negative likelihood ratio (NLR) of aqueous humor, when cut off values are the same with those that used for the serum.

	*L. infantum*	*T. gondii*	*A. phagocytophilum*	*B. caballi*
Sensitivity (%)	33.33	25.00	30.77	28.57
95% CI	0.84–90.57	0.63–80.59	9.09–61.43	3.67–70.96
Specificity (%)	100.00	100.00	100.00	100.00
95% CI	94.79–100.00	94.72–100.00	93.94–100.00	94.48–100.00
PLR	-	-	-	-
95% CI	-	-	-	-
NLR	0.67	0.75	0.69	0.71
95% CI	0.30–1.48	0.43–1.32	0.48–0.99	0.45–1.14

PLR: positive likelihood ratio, NLR: negative likelihood ratio, CI: confidence interval.

**Table 4:** Sensitivity, specificity, positive likelihood ratio (PLR) and negative likelihood ratio (NLR) of aqueous humor, when cut off value is 1:10.

	*L. infantum*	*T. gondii*	*A. phagocytophilum*	*B. caballi*
Sensitivity (%)	60.00	25.00	38.46	28.57
95% CI	14.66–94.73	0.63–80.59	13.86–68.42	3.67–70.96
Specificity (%)	100.00	100.00	98.31	98.46
95% CI	94.79–100.00	94.72–100.00	90.91–99.96	91.72–99.96
PLR	-	-	22.69	18.57
95% CI	-	-	2.89–178.29	1.92–179.82
NLR	0.40	0.75	0.63	0.73
95% CI	0.14–1.17	0.43–1.32	0.41–0.96	0.45–1.16

PLR: positive likelihood ratio, NLR: negative likelihood ratio, CI: confidence interval.

## Data Availability

The data presented in this study are available on request from the corresponding author. The data are not publicly available due to further processing for other studies.

## Supplementary Materials

The following are available online at https://www.mdpi.com/article/10.3390/microorganisms9122616/s1, Table S1: Results of antibody detection against *L. infantum*, *T. gondii*, *A. phagocytophilum* and *B. caballi* in wild rabbits, in serum and in aqueous humor at individual level using different cut-off points.
